# COVID-19 in Greece: People take precedence over economics

**DOI:** 10.7189/jogh.10.020337

**Published:** 2020-12

**Authors:** Thomas J Papadimos

**Affiliations:** Critical Care Section, Department of Anesthesiology, The Ohio State University Wexner Medical Center, Columbus, Ohio, USA

The Greek minister of state, Giorgos Gerapetritis, told the New York Times [1]: “*We acted pre-emptively. We consciously preferred to make a significant financial sacrifice rather than sacrifice human life.*”

The tiny country of Greece has 10.7 million people. It endured 400 years of Ottoman domination, the Balkan wars in the early 20^th^ century, devastation by the Nazis in World War II, the Greek civil war of 1947-1949, the military junta of 1967-1974, and a crippling ten-year economic crisis that was just about over when COVID-19 reared its ugly head [2]. To make things even more difficult Greece’s elderly population is only second to Italy in the European Economic Union [3].

COVID-19 made its appearance in Greece on February 26, 2020 in a young Greek woman who had just returned from Italy. The next day all festivals in the country were cancelled. Locally affected jurisdictions took action in early March to close schools and other cultural events. On March 10 all educational institutions were closed; on March 13 all restaurants, cafes, bars, museums, shopping malls, and sports venues were closed; on March 16 all retail shops were closed; and on March 18 the government made available 10 billion euros to support employees, businesses, and the economy. Furthermore, on March 23 movement was restricted, every citizen needed a signed attestation for the reason of their movement, and they were required to carry their passport or national identity card during any transit. National and local police, the Hellenic Coast Guard, and the National Transportation Authority enforced the orders and issued fines for violations by citizens or businesses. Borders were closed and there were restrictions on trips to the Greek islands. All restrictions were extended to May 4th [4-6]. All visitors were required to quarantine for 14 days or face a $5400 fine and possible expulsion [1]. The ban on all educational institutions and large social gatherings occurred before even the first death was reported [7]. And now, as the country tries to reopen, it may restrict tourism to those countries who have been able to responsibly control COVID-19 [8].

The financial crisis of 2009-2018 required severe cost-cutting measures that resulted in a 60% cut to the public and private health sectors. By 2017 the Greek health care system was declared to be in a meltdown [7]. How could a country this small deal with the COVID-19 crisis that was upon them with only 565 intensive care unit (ICU) beds? Something extraordinary happened in their politics. The Mitsotakis government decided to listen to the scientists. Dr Sotirios Tsiodras, an infectious disease specialist at the University of Athens, was made the government spokesperson. Through philanthropic, public, and private collaborations, the ICU beds were increased to 910 (a 62% increase) by the end of March, 3337 additional staff members were hired in the national health system, and another 942 physicians were scheduled to be hired [1]. Greece rose to the occasion in response to its public health needs [9].

**Figure:**
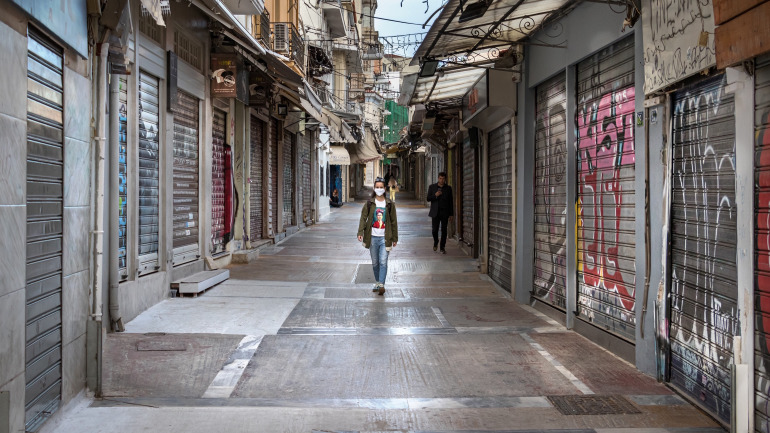
Photo: The empty streets of Athens, Greece during lockdown (courtesy of Dreamstime.com).

Dr Tsiodras held a briefing every evening at 1800 hours on national television and presents the most current scientific findings on COVID-19, and he was followed by the deputy minister for civil protections and crisis management, Nikos Chardalias, regarding any new civil actions that may be required. These two gentlemen present the latest global and national information that is available from that day thereby interdicting any fake news, disinformation, or misinformation. Here, science and policy decisions intersect. The Greek populace watches them religiously every evening. They have become the two most respected and popular figures in Greece.

While these efforts are laudable, this health crisis will result in a financial crisis of consequential proportions for the Greek economy. In Europe this will particularly effect countries that elected to implement austerity measures (such as Greece, Spain, and Italy) [10]. Governments and their central banks will intervene through lowering interest rates and/or the provision of fiscal stimuli. These measures will probably only provide limited remission of the circumstances. Protecting lives is of the utmost importance in the preservation and recovery of an economy [10]. In the aftermath of COVID-19 countries will experience more suicides, alcoholism and drug-related mortalities, as well as the need for emergency food support, with an accompanying decrease in life expectancy. Furthermore, in not showing a concern for the protection of its citizens, a government may see a change in the number of citizens who can pay taxes, voting attitudes, voting patterns, and an electorate leaning towards the more extreme elements in politics. The Greek government’s emphasis on human life over profit has united the country, and hopefully has laid a foundation that will keep the country united, avoid unrest, and the above-mentioned illnesses of despair.

Greece has seen many troubles, and the country was determined not want to have the same fate as their Spanish and Italian neighbors in regard to COVID-19, realizing that another serious calamity would be another unacceptable catastrophe, both in financial and human terms. The populace recognized the importance of complying with their government’s instructions. The Greeks have become experts through their history, recent and past, at weathering crises. Nonetheless, the politics in Greece can be just as complicated as in any other country. The Greek government, through Tsiodras and Chardalias, sent a persistent and crystal-clear message, the economy was very important, but it came second to human lives and physical suffering.

In this critical moment in the world’s history, that small country which gave us philosophy, democracy, science, and ethics demonstrated its maturity as opposed to some of its younger counterparts of the West. If you have a good grasp of the world’s history in regard to suffering, and have suffered yourself, your view of COVID-19 becomes quite different regarding a stay-at-home order or wearing a mask in public. If scientific leaders and politicians take the opportunity to speak honestly, openly, often, and send a consistent message, much can be accepted and accomplished by a citizenry. I do not know what the COVID-19 future holds for Greece, but they approached this initial crisis in an exemplary manner. We should all consider following their lead of consistent messaging, evidence-based evaluation, and adherence to the scientific method.
